# Agonistic anti-ICAM-1 antibodies in scleroderma: Activation of endothelial pro-inflammatory cascades

**DOI:** 10.1016/j.vph.2013.05.002

**Published:** 2013-07

**Authors:** Sabine I. Wolf, Sarah Howat, David J. Abraham, Jeremy D. Pearson, Charlotte Lawson

**Affiliations:** a Department of Comparative Biomedical Sciences, The Royal Veterinary College, London, UK; b Cardiovascular Division, School of Medicine, King's College London, UK; c Centre for Rheumatology and Connective Tissue Disease, University College London Medical School, Royal Free Campus, London, UK

**Keywords:** Systemic sclerosis, ICAM-1, Autoantibodies, Vascular inflammation

## Abstract

**Background:**

Scleroderma (SSc) is a complex autoimmune disorder that can be characterised by the presence 2of circulating autoantibodies to nuclear, cytoplasmic and cell surface antigens. In particular antibodies directed against endothelial cell antigens (anti-endothelial cell antibodies; AECA) have been detected.

ICAM-1 is an adhesion molecule expressed on the surface of human endothelial cells. We have previously shown that cross-linking ICAM-1 with monoclonal antibodies leads to pro-inflammatory activation of human endothelial and vascular smooth muscle cells and that cardiac transplant recipients with transplant associated vasculopathy make antibodies directed against ICAM-1.

**Objectives:**

To determine whether SSc patients make antibodies directed against ICAM-1 and whether these antibodies induce pro-inflammatory activation of human endothelial cells in vitro.

**Methods:**

Using recombinant ICAM-1 as capture antigen, an ELISA was developed to measure ICAM-1 antibodies in sera from SSc patients. Antibodies were purified using ICAM-1 micro-affinity columns. HUVEC were incubated with purified anti-ICAM-1 antibodies and generation of reactive oxygen species, and expression of VCAM-1 was measured.

**Results:**

Significantly elevated levels of anti-ICAM-1 antibodies were detected in patients with diffuse (dSSc; 10/31 32%) or limited (lSSc; 14/36 39%) scleroderma. Cross-linking of HUVEC with purified anti-ICAM-1 antibodies caused a significant increase in ROS production (2.471 ± 0.408 fold increase above untreated after 150 min p < 0.001), and significant increase in VCAM-1 expression (10.6 ± 1.77% vs 4.12 ± 1.33%, p < 0.01).

**Conclusion:**

AECA from SSc patients target specific endothelial antigens including ICAM-1, and cause pro-inflammatory activation of human endothelial cells, suggesting that they are not only a marker of disease but that they contribute to its progression.

## Graphical abstract

**Figure:**
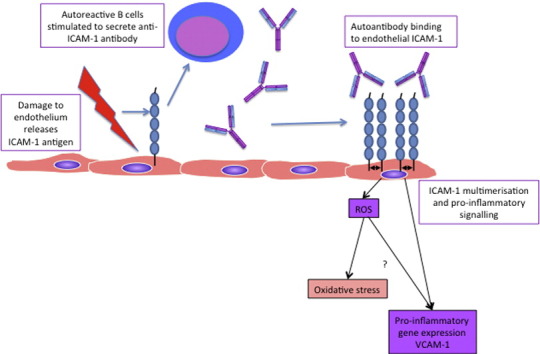

## 1 Introduction

### 1.1 Scleroderma and autoantibodies

Scleroderma or systemic sclerosis (SSc), is an autoimmune connective tissue disorder that targets fibroblasts and the vascular endothelium. Limited SSc (lSSc) affects mainly the skin of the hands, face and arms, although pulmonary arterial hypertension may also be a serious complication. Diffuse SSc (dSSc) is the rapidly progressing form of the disease, characterised by severe fibrosis of large areas of skin and visceral organs, as well as widespread vascular injury (Kao and Weyand, 2010). Although the underlying pathologic triggers remain elusive, there is evidence for expansion of circulating B cells in SSc patients (Sato et al., 2004), altered T cell responses leading to a Th2 cytokine milieu (Gabrielli et al., 2007; Hasegawa et al., 1997; Hasegawa et al., 1998), and there are many reports describing the presence of autoantibodies in serum from these patients (review (Mihai and Tervaert, 2010)).

Anti-endothelial cell antibodies (AECA) were first described in the literature more than 40 years ago (review (Gabrielli et al., 2007)) in sera from patients with a number of different rheumatic diseases. AECA appear to recognise a number of different endothelial epitopes, including cell surface, cytoplasmic and nuclear antigens, and there is increasing evidence that at least some species may have agonistic properties i.e. they appear to cause endothelial cell activation and therefore are hypothesised to contribute to disease progression.

### 1.2 Agonistic anti-ICAM-1 antibodies

ICAM-1 is a 90 kDa Ig superfamily protein, expressed on the surface of several cell types including endothelial cells, where it has been shown to be a critical molecule for firm adhesion and trans-endothelial migration of several leukocyte subsets. ICAM-1 itself can activate cell signalling cascades after receptor multimerisation which can be achieved in vitro by co-culturing of EC with LFA-1 positive leukocytes, artificial clustering of ICAM-1 on EC by fibrinogen, or cross-linking with anti-ICAM-1 antibodies (review (Hubbard and Rothlein, 2000; Lawson and Wolf, 2009)).

A number of signalling molecules and adapter proteins have been linked with the ICAM-1 signalling cascade in vitro, depending on cell lineage or origin of vascular bed of EC used, and the experimental model (for review see (Lawson and Wolf, 2009)). Cross-linking of ICAM-1 on the cell surface of endothelial cells leads to its redistribution from the detergent soluble to insoluble fraction, suggesting that it is involved with endothelial cytoskeletal rearrangements required for leukocyte emigration (Amos et al., 2001). A number of studies have also demonstrated that ICAM-1 cross-linking, either with monoclonal antibodies or during co-culture of endothelial cells with T cells, leads to activation of molecules involved in these rearrangements including Rho-A, a small GTPase responsible for actin stress fibre formation (for review see (Lawson and Wolf, 2009)).

In addition to its role in actin cytoskeleton rearrangements, cross-linking of ICAM-1 with monoclonal antibodies has also been shown to activate pro-inflammatory cascades, via activation of MAPK kinases ERK-1/2 and/or JNK (Etienne et al., 1998; Lawson et al., 1999; Sano et al., 1998). Activation of ERK-1 leads to AP-1 activation (Lawson et al., 1999) and ERK-dependent production and secretion of IL-8 and RANTES (Sano et al., 1998), expression of VCAM-1 expression on the cell surface (Lawson et al., 1999; Lawson et al., 2001). ICAM-1 cross-linking also upregulates tissue factor production (Schmid et al., 1995) and expression of proinflammatory cytokines including IL-1β (Koyama et al., 1996).

We have previously identified agonistic anti-ICAM-1 antibodies in serum from cardiac transplant recipients with chronic transplant vasculopathy and shown that serum from these patients can activate phosphorylation of p42/p44 Erk MAPK (Lawson et al., 2005). In the present study we have identified anti-ICAM-1 antibodies present in serum from patients with SSc significantly above levels seen in healthy volunteers. Using microaffinity columns we have purified IgG anti-ICAM-1 antibodies from these patients and shown that they bind to human umbilical vein endothelial cells (HUVEC) and activate reactive oxygen species production and VCAM-1 expression.

## 2 Methods

### 2.1 Materials

All chemicals were from Sigma (Poole, Dorset, UK) unless otherwise stated. All plastics and tissue culture plates and flasks were from Nunc (Fisher Scientific, Loughborough, UK). All tissue culture consumables were from PAA (UK) except for endothelial growth supplement (ECGS) from Sigma. Anti-VCAM-1 MAb clone 1.4C3 and anti-ICAM-1 MAb clone 6.5B5 were gifts from Professor Dorian Haskard (Imperial College, London). Secondary FITC or HRP conjugated antibodies were from DAKO. Rabbit anti-mouse Ig (RAM) used to cross-link 6.5B5 was from DAKO. Rabbit anti-human Ig (RAH) used to cross-link patient antibodies was from Jackson Immunotech (Stratech, Luton, UK). Recombinant human TNF was from Insight Biotechnology (Wembley, London, UK).

### 2.2 Source of human sera

Sera were collected from 60 patients with scleroderma (n = 32 lSSc, n = 28 dSSc) and from 26 healthy controls. Sera were collected at the Royal Free Hospital; Centre for Rheumatology, London, UK; with the informed consent of the patient. Volunteers with SSc fulfilled the criteria of the ACR for the diagnosis of SSc and ethical approval was obtained from the Royal Free Hospital ethical practices sub-committee.

### 2.3 Preparation of recombinant ICAM-1

Recombinant human ICAM-1 was cloned into pET15b (Novagen, MerckMillipore, Darmstadt, Germany) and purified from cultures of BL21 *E. coli* using His-bind columns as described previously (Holder et al., 2008).

### 2.4 ELISA

Purified recombinant human ICAM-1 (rhICAM-1) was coated onto alternate rows of 96 well Maxisorp ELISA plates (Nunc, Fisher Scientific, Loughborough, UK) at a concentration of 2 μg per well in 150 μl 1 × PBS. The remaining wells were coated with 1 × PBS alone to measure non-specific binding of Ig to the microtitre plate. Plates were incubated at 4 °C overnight. The following morning plates were washed 3 times in 1 × PBS 0.1% Tween-20 (PBST) and all wells were blocked for 1 h with 300 μl 5% non-fat milk powder PBS-T (Marvel-PBST), followed by three washes in PBST. Patient samples were diluted 1:50 in Marvel-PBST and added to rhICAM-1 coated and PBS coated wells in triplicate at 100 μl/well. Samples were incubated for 2 h at room temperature followed by three washes in PBST. Positive controls were monoclonal anti-ICAM-1 6.5B5 at 10, 5, 2.5, 1.25 μg/ml (10 μg/ml was used for calculation of ELISA RATIO, see below) and negative controls were Marvel-PBST alone. Wells were incubated with 100 μl HRP-conjugated rabbit-anti-human-IgG or IgM or HRP-conjugated-rabbit-anti-mouse IgG as appropriate and incubated for 45 min at room temperature, followed by three washes in PBST. 100 μl TMB supersensitive substrate (Sigma) was added and plates were incubated for 5 min at room temperature before the reaction was stopped by addition of 50 μl 1 M H_2_SO_4_. Plates were read using a Wallac 1410 plate reader at 450 nm. In order to normalise OD results across multiple plates and with multiple batches of recombinant ICAM-1, an “ELISA ratio” (ER) was used which was calculated according to the equation:

$$\mathrm{ER}=\frac{\left[{\text{OD}}_{450}\text{serum}\;\text{Ig}\;\text{bound}\;\text{to}\;\text{ICAM1}\;\text{coated}\;\text{wells}]-[{\text{OD}}_{450}\text{serum}\;\text{Ig}\;\text{bound}\;\text{to}\;\text{PBS}\;\text{coated}\;\text{wells}\right]}{\left[{\text{OD}}_{450}\;6.5B5\;\left(10\;\mathrm{\mu g}/\mathrm{ml}\right)\text{to}\;\text{ICAM1}\;\text{coated}\;\text{wells}]-[{\text{OD}}_{450}\;6.5\text{B}5\left(10\;\mathrm{\mu g}/\mathrm{ml}\right)\text{bound}\;\text{to}\;\text{PBS}\;\text{coated}\;\text{wells}\right]}\text{.}$$

Sera were deemed to contain a high titre of anti-ICAM-1 antibodies if the ER was greater the mean ER + 3sd of the negative control sera.

### 2.5 Purification of ICAM-1 antibodies from human serum

Recombinant hICAM-1 was prepared as described above and coupled to Pierce micro-affinity columns according to the manufacturer's instructions (Fisher Scientific). Anti-ICAM-1 antibodies were purified from 200 μl SSc patient serum (n = 6 patients) selected as highly positive for anti-ICAM-1 as determined by our ELISA, and the manufacturer's instructions were followed. All washes were kept for use as negative controls. Purified antibodies were analysed using the anti-ICAM-1 ELISA described above to determine the relative concentrations compared to the 6.5B5 positive control anti-ICAM-1 MAb, to confirm their presence (data not shown).

### 2.6 HUVEC isolation and culture

Human umbilical cords were collected from the Royal London Hospital with ethical approval from the East London Research Ethics Committee. Human umbilical vein endothelial cells (HUVEC) were isolated using a modified method of Jaffe and maintained in HUVEC medium (M199 supplemented with 20% FCS, 2 mM l-glutamine, 1 × penicillin/streptomycin, 30 μg/ml ECGS from bovine neural tissue and 1 unit/ml heparin) on 0.5% gelatin-coated tissue culture plates as described previously (Lawson et al., 1999). Individual isolates of cells were used at passage 3–5.

### 2.7 Analysis of binding of purified antibodies to HUVEC by flow cytometry

HUVEC were plated onto gelatin coated 6 well plates at a density of 2.5 × 10^5^ cells per well. The following day the medium was removed and replaced with M199/10% FCS for 4 h. After this time medium was removed and replaced with M199/10% FCS containing purified anti-ICAM-1 antibody, negative serum, wash buffer from antibody purification (all at 1:100 dilution), 10 ng/ml TNF or RAH Ig as negative controls. Cells were incubated for 18 h. The following day cells were trypsinised and washed with 1 × PBS followed by centrifugation at 405 x*g* to remove unbound antibodies. Cell pellets were resuspended in 50 μl 1 × PBS with diluted FITC-conjugated goat-anti-human IgG (1:100 from manufacturers stock), incubated for 30 min at 4 °C followed by a further wash with 1 × PBS and centrifugation at 405 x*g*. Cell pellets were resuspended in 500 μl 0.5%formaldehyde/1 × PBS and analysed by flow cytometry using a FACS CANTO II. Post-acquisition analysis was carried out using FACS DIVA software.

### 2.8 Measurement of reactive oxygen species (ROS) generation in HUVEC after ICAM-1 cross-linking by purified anti-ICAM-1 antibodies

HUVEC were plated onto gelatin-coated white opaque 96 well plates at a density of 2.5 × 10^4^ cells per well in full HUVEC medium with growth factor. The following day medium was removed and replaced with M199/10%FCS for 4 h. After this the medium was removed from cells and replaced with 50 μl medium containing 5.7 μM dihydrorhodamine-1,2,3 (DHR), which acts as an indicator of reactive oxygen species (ROS) production. DHR is taken up by live cells and is not fluorescent until it becomes oxidised in the presence of intracellular peroxidases and intracellular H_2_O_2_ (Henderson and Chappell, 1993). Cells were incubated for 10 min in the dark and then treated with 50 μl purified anti-ICAM-1 antibody, whole fraction of positive serum, washes from the purification columns, or whole fraction of negative serum as appropriate (all diluted 1:100 in M199/10% FCS), for 10 min. After 10 min incubation the cross-linking rabbit-anti-human Ig antibody (RAH; dilution 1:200; Jackson, Stratech, UK) was added as appropriate (or 50 μl M199/10%FCS for non-cross-linked wells) and plates were incubated at 37 °C. Fluorescence was measured at t = 0, 10, 30, 60, 150 min using a Wallac 1410 fluorescent plate reader (FITC filter).

All treatments were carried out in triplicates using n = 2–6 separate isolates of HUVEC to test n = 6 purified anti-ICAM-1 antibodies, n = 6 whole serum, n = 3 negative serum, n = 3 wash from n = 3 separate purifications.

### 2.9 Analysis of VCAM-1 expression on HUVEC by flow cytometry, after ICAM-1 cross-linking by purified anti-ICAM-1 antibodies

HUVEC were plated onto gelatin coated 6 well plates at a density of 2.5 × 10^5^ cells per well. The following day the medium was removed and replaced with M199/10% FCS for 4 h. After this time medium was removed and replaced with M199/10% FCS containing purified anti-ICAM-1 antibody, negative serum, wash buffer from antibody purification or monoclonal 6.5B5 as appropriate. Cells were incubated for 30 min at 37 °C before the medium was removed and replaced with medium containing rabbit anti-human IgG (RAH; 1:200 dilution; Jackson, Stratech, UK) or rabbit anti-Mouse IgG (RAM; 1:200 dilution Dako, UK) to cross-link cells for 18 h (6 h to 24 h for time course experiment). Incubation of HUVEC with 10 ng/ml rhTNF for 18 h was used as a positive control. The following day cells were trypsinised and washed with 1 × PBS followed by centrifugation 405 x*g* to pellet cells. Cell pellets were resuspended in 50 μl 1 × PBS with diluted anti-VCAM-1 MAb 1.4C3 as primary antibody, (or with CRL1724 as a negative control; n = 2) incubated for 30 min at 4 °C and washed as above. Cell pellets were resuspended in 50 μl FITC-conjugated goat-anti-human IgG (1:100 from manufacturer's stock), incubated for 30 min at 4 °C and washed as above. Cell pellets were resuspended in 500 μl 0.5%formaldehyde/1 × PBS and analysed by flow cytometry using a FACS CANTO II. Post-acquisition analysis was carried out using FACS DIVA software.

### 2.10 Statistical analysis

Graphpad Prism was used to analyse data. Data were analysed for normal distribution using the D'Agostino–Pearson Omnibus normality test. For normally distributed data 1 or 2 way ANOVA followed by Bonferroni post-tests were used as appropriate. For non-parametric data Mann–Whitney tests, Kruskal–Wallis ANOVA (unpaired data) or a Friedman ANOVA (paired/repeated measures data) were performed, followed by Dunns post-comparison tests. p < 0.05 was considered statistically significant (*). Data are presented as mean + SEM.

## 3 Results

### 3.1 Detection of antibodies to ICAM-1 in serum from SSc patients

**Fig. 1:**
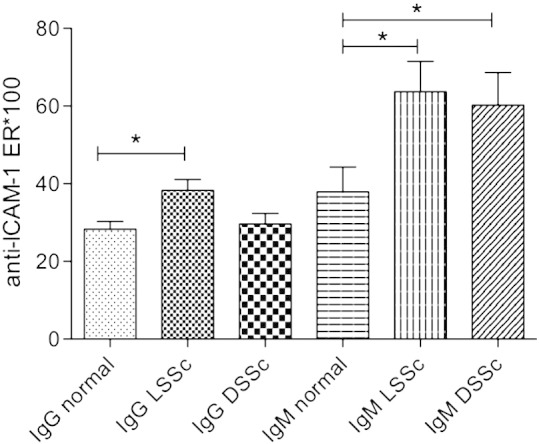
Detection of anti-ICAM-1 antibodies in serum from SSc patients. Levels of anti-ICAM-1 IgG and IgM antibodies were measured in serum from 60 patients with scleroderma (n = 32 LSSc, n = 28 DSSc) and from 26 healthy controls, using immobilised recombinant human ICAM-1 as the capture antigen. *p < 0.05; **p < 0.01, Mann–Whitney tests compared to healthy controls.

The presence of immunoglobulins (IgM or IgG) able to bind to immobilised recombinant human ICAM-1 was analysed in serum from 60 patients with scleroderma (n = 32 lSSc, n = 28 dSSc) and from 26 healthy controls. Significantly elevated levels of anti-ICAM-1 antibodies were detected in 10/31 (32%) patients with dSSc and 14/36 (39%) patients with lSSc, compared to normal control sera. As shown in Fig. 1 levels of IgM against ICAM-1 were significantly increased compared to normal controls in both lSSc and dSSc patients (0.693 ± 0.082 lSSc; dSSc ER = 0.664 ± 0.093, both p < 0.01 compared to normal controls ER 0.397 ± 0.075). The levels of anti-ICAM-1 of IgG subclass were also increased in lSSc patients compared to normal controls (ER 0.2781 ± 0.02 normal; 0.3962 ± 0.02 LSSc, p < 0.001 compared to normal controls). Levels of anti-ICAM-1 IgG were not significantly enhanced in dSSc patients compared to normal controls (ER 0.3022 ± 0.03).

### 3.2 Anti-ICAM-1 antibodies purified from SSc patients bind to HUVEC cell surface

**Fig. 2:**
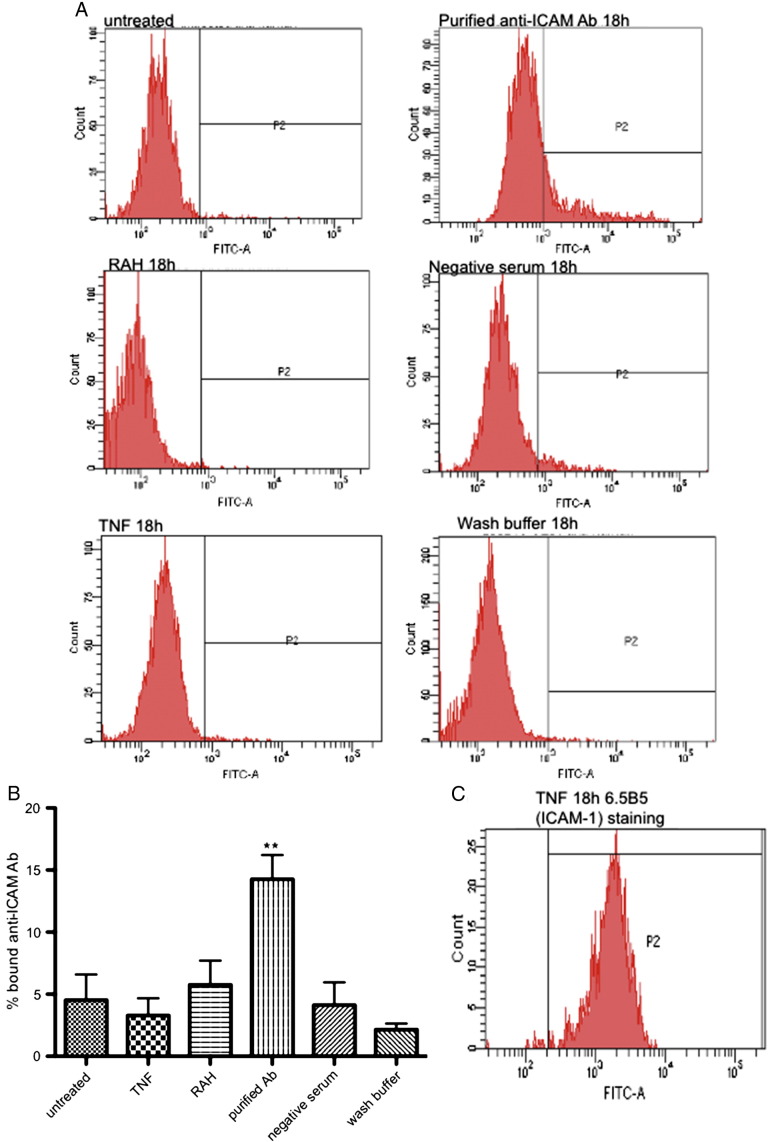
Purified anti-ICAM-1 antibodies bind to human endothelial cells. Anti-ICAM-1 antibodies were purified from SSc patients using microaffinity columns and incubated with HUVEC for 18 h at 37 °C, followed by confirmation of their binding with a FITC-conjugated anti-human-IgG and analysis by flow cytometry. As negative controls HUVEC were additionally incubated with negative serum (diluted 1:100), wash buffer from the microaffinity purification (diluted 1:100), rabbit-anti-human IgG (unconjugated; RAH) or 10 ng/ml TNF, to confirm that increase in fluorescence was not due to non-specific binding via endothelial FcR. A. Representative histograms showing FITC staining. P2 denotes FITC positive events. B. Graphs to show binding of purified antibodies from n = 6 patients and controls. C. Representative histogram to show ICAM-1 expression after incubation of HUVEC with 10 μg/ml TNF for 18 h followed by staining with 6.5B5 anti-ICAM-1 mouse monoclonal antibody and rabbit-anti-mouse-Ig-FITC (n = 2). **p < 0.01 binding of purified Ab compared to all other overnight incubations, 1 way ANOVA followed by Bonferroni post-test.

Anti-ICAM-1 antibodies were purified by microaffinity chromatography from serum from six SSc patients with high reactivity to ICAM-1, and binding of purified IgG to recombinant ICAM-1 in the purified preparations was reaffirmed by anti-ICAM-1 ELISA (data not shown). The ability of the purified anti-ICAM-1 antibodies to bind to HUVEC cell surface was confirmed by flow cytometry (Fig. 2A). As shown in Fig. 2B FITC-anti-human-IgG bound to 14.26 ± 1.95% of cells that had been incubated for 18 h with anti-ICAM Ab purified from SSc patients. In contrast, there was only binding of FITC-anti-human-IgG to 4.53 ± 2.07% untreated cells (p < 0.01) or 5.75 ± 1.96% cells pretreated with only the rabbit anti-human IgG cross-linking antibody (RAH) (p < 0.05). In addition, using only anti-Human-IgF FITC we did not detect any increase in fluorescence of HUVEC which had been incubated for 18 h with the whole fraction of serum previously shown to be negative on our ICAM-1 ELISA (FITC-anti-human-IgG bound to 4.12 ± 1.83% cells), the wash buffer used during the affinity purification process (FITC-anti-human-IgG bound to 2.16 ± 0.48% cells), or cells which had been treated overnight with 10 ng/ml TNF (3.3 ± 1.4%). To confirm that TNF was able to increase ICAM-1 expression we stained TNF treated HUVEC with anti-ICAM-1 6.5B5 followed by rabbit-anti-mouse-Ig-FITC (Fig. 2C). These findings confirm that the purified anti-ICAM-1 antibody bound to a specific cell surface receptor rather than non-specific binding of the FITC-conjugated secondary anti-human-Ig to endothelial Fc receptors.

### 3.3 Cross-linking of purified anti-ICAM-1 antibodies from SSc patients leads to reactive oxygen species generation in HUVEC

**Fig. 3:**
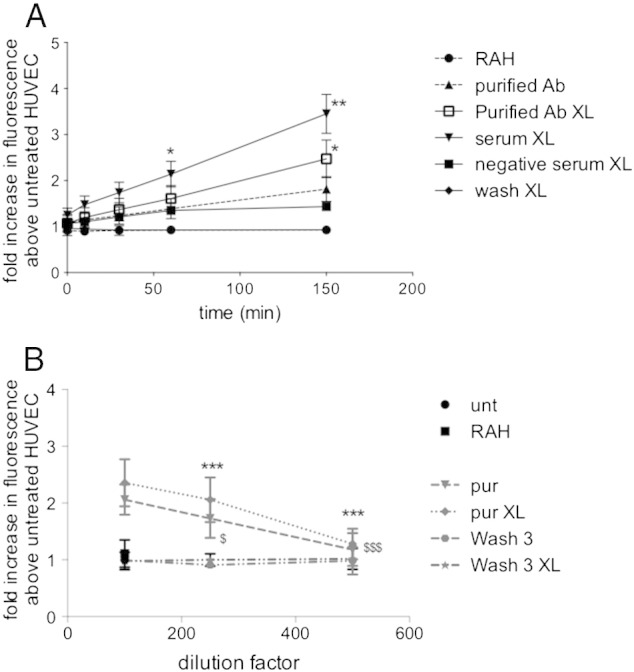
Purified anti-ICAM-1 antibodies induce reactive oxygen species production in human endothelial cells. A. Purified anti-ICAM-1 antibodies (diluted 1:100), in the presence or absence of crosslinking RAH antibody, were incubated with HUVEC had been pre-loaded with dihydrorhodamine-1,2,3. Fluorescence was measured at regular intervals up to 150 min. As negative controls HUVEC were treated with RAH alone, negative serum (diluted 1:100) and RAH or wash buffer and RAH. *p < 0.05 ROS production after incubation with purified ICAM-1 antibody and **p < 0.01, ***p < 0.001 ROS production after cross-linking of purified ICAM-1 antibody, compared to RAH alone incubations, 2 way ANOVA with Bonferroni post-test (antibodies purified from n = 6 patients). B. Purified anti-ICAM-1 antibodies, in the presence or absence of crosslinking RAH antibody, were incubated with HUVEC had been pre-loaded with dihydrorhodamine-1,2,3 at dilutions of 1:100, 1:250 or 1:500 fluorescence was measured at 150 min. 2 way ANOVA with Bonferroni post-test ^$^p < 0.05 ROS production after incubation with purified ICAM-1 antibody at 1:100 dilution compared to 1:250 dilution and ^$$$^p < 0.001 compared to 1:500 dilution; ***p < 0.001 ROS production after cross-linking of purified ICAM-1 antibody at 1:100 dilution compared to 1:250 or 1:500 dilution (antibodies purified from n = 4 patients).

Purified anti-ICAM-1 antibodies were allowed to bind to dihydrorhodamine-1,2,3 loaded HUVEC followed by cross-linking with RAH. Fluorescence was measured at regular intervals after initiation of cross-linking. Significant production of ROS was induced after cross-linking HUVEC with the whole fraction of positive serum (3.45 ± 0.424 fold increase above untreated cells after 150 min p < 0.001) or purified anti-ICAM-1 antibodies (2.471 ± 0.408 fold increase above untreated after 150 min p < 0.001), when compared to cells treated with RAH alone (0.927 ± 0.076 fold increase above untreated cells). In contrast, there was no significant increase in ROS production above RAH alone treated cells in cells cross-linked with negative serum (1.437 ± 0.169) or with the wash fraction (0.93 ± 0.016) (Fig. 3A). To determine whether there was a concentration dependent effect of antibody binding on ROS production, increasing dilutions of purified patient anti-ICAM-1 antibodies (1:100, 1:250, 1:500) or wash fraction at similar dilutions was incubated with DHR-loaded HUVEC for 150 min (Fig. 3B). ROS production decreased significantly with increased dilution of antibody (2.060 ± 0.133 fold increase in ROS at 1:100 dilution compared to 1.729 ± 0.170 at 1:250 p < 0.05; and 1.18 ± 0.145 at 1:500 p < 0.001). This was also the case after crosslinking with RAH (2.2356 ± 0.207 at 1:100 dilution compared to 2.058 ± 0.196 at 1:250 dilution p < 0.001 and 1.280 ± 0.134 at 1:500 dilution p < 0.001) (Fig. 3B).

### 3.4 Cross-linking of purified anti-ICAM-1 antibodies from SSc upregulates VCAM-1 expression in HUVEC

**Fig. 4:**
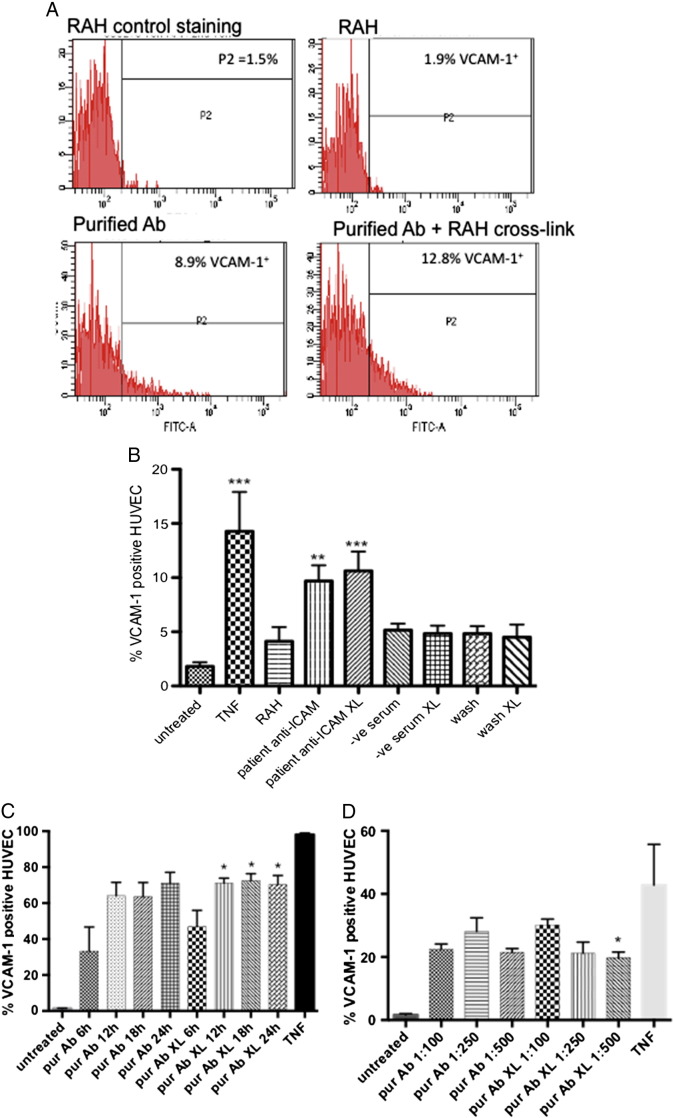
Purified anti-ICAM-1 antibodies induce VCAM-c1 expression on human endothelial cells. Purified anti-ICAM-1 antibodies, in the presence or absence of crosslinking RAH antibody, were incubated with HUVEC for 18 h at 37 °C followed by indirect immunofluorescence staining for VCAM-1 and analysis by flow cytometry. A. Representative (n = 6) histograms for staining with CRL1724 isotype control, RAH (negative control) treated cells, and cells treated with purified anti-ICAM-1 (diluted 1:100) with or without crosslinking. B. Percentage of VCAM-1 positive HUVEC from n = 6 experiments after treatment with TNF (positive control), negative serum (diluted 1:100), wash buffer (diluted 1:100) with or without RAH cross-linking (negative controls), or purified patient anti-ICAM-1 antibodies (diluted 1:100) with or without crosslinking. **p < 0.01, ***p < 0.001 compared to untreated cells. 1 way ANOVA with Bonferroni post-test. Antibodies purified from n = 6 patients. C. Time course of VCAM-1 expression after treatment with purified patient anti-ICAM-1 antibodies (diluted 1:100) with or without RAH cross-linking for 6, 12, 18, 24 h (n = 4), or VCAM-1 staining after TNF treatment 18 h (n = 2 HUVEC isolates) (Friedman ANOVA for non-parametric data followed by Dunns post-test comparison *p < 0.05 crosslinked purified Ab 6 h compared to crosslinked purified Ab 12 h, 18 h, or 24 h). D. Concentration effect of purified patient anti-ICAM-1 antibodies (diluted 1:100; 1:250; 1:500) with or without RAH cross-linking on VCAM-1 expression in HUVEC or VCAM-1 staining after TNF treatment 18 h (n = 2 HUVEC isolates) (Friedman ANOVA for non-parametric data followed by Dunns post-test comparison *p < 0.05 crosslinked purified Ab diluted 1:100 compared to crosslinked purified Ab diluted 1:500).

Purified anti-ICAM-1 antibodies (1:100 dilution) were allowed to bind to HUVEC, followed by cross-linking with RAH for between 6 and 24 h. HUVEC were trypsinised and VCAM-1 expression was determined by flow cytometry (Fig. 4A). There was a significant increase in VCAM-1 cell surface staining after incubation with purified anti-ICAM-1 antibodies for 18 h with or without cross-linking, compared to treatment of cells with RAH alone (9.69 ± 1.46% on anti-ICAM-1 bound cells; 10.6 ± 1.77% on anti-ICAM-1 cross-linked cells vs 4.12 ± 1.33% on RAH alone treated cells, p < 0.01 for each; 1 way ANOVA with Bonferroni post-test), however no increase was observed after cross-linking with negative serum or with the wash fraction (4.85 ± 0.73% and 4.51 ± 1.16% respectively) (see Fig. 4A for representative plots; Fig. 4B for averaged data). In a separate experiment to determine the time course of VCAM-1 expression after ICAM-1 cross-linking with purified antibodies, separate isolates HUVEC were incubated for between 6 h and 24 h before staining for VCAM-1 expression. VCAM-1 was elevated after 6 h incubation with purified patient antibodies and remained significantly elevated for at least 24 h compared to HUVEC treated with the wash fraction alone, with or without cross-linking with RAH (p < 0.001 Friedman Test for non-parametric data and p < 0.05 Dunns post-test comparison crosslinked purified Ab 6 h compared to crosslinked purified Ab 12 h, 18 h, or 24 h; Fig. 4C). In a further experiment addition of decreasing concentrations of purified antibodies (1:100, 1:250, 1:500) significantly altered the level of VCAM-1 expression after 18 h incubation (p < 0.05 Friedman repeated measures ANOVA for non-parametric data and p < 0.05 Dunns post-test comparison crosslinked purified Ab diluted 1:100 compared to crosslinked purified Ab diluted 1:500; Fig. 4D).

## 4 Discussion

Here, we have developed a novel ELISA to detect the presence of antibodies directed against ICAM-1 in the serum of patients with limited and diffuse forms of SSc. We have purified anti-ICAM-1 antibodies from serum and shown that they can induce pro-inflammatory signalling and gene expression in human endothelial cells in vitro.

Antibodies directed against cell surface antigens on endothelial cells (AECA) have been described in a number of autoimmune diseases, including rheumatoid arthritis (Salih et al., 1999), lupus (Constans et al., 2003), vasculitis (Salih et al., 1999), and SSc (Carvalho et al., 1996). There is also a large literature on the presence of antibodies directed against cytoplasmic or nuclear antigens in these patients (Cavazzana et al., 2009; Moroi et al., 1980), some of which also appear to be against endothelial antigens (Ahmed et al., 2006). AECA have likewise been detected in patients with atherosclerosis (Farsi et al., 2001) and those with vascular rejection of heart transplants (Fredrich et al., 1999). AECA themselves have been shown to be damaging to endothelial cells in vitro (Alessandri et al., 2006; Xia and Kellems, 2011) including pro-apoptotic signalling (Ahmed et al., 2006; Hernandez et al., 2012; Holmen et al., 2007). A number of studies have also shown associations between the presence of AECA and increased morbidity, mortality or cardiovascular complications, suggesting that the antibodies are agonistic and contribute to endothelial dysfunction that is often associated with autoimmune and chronic inflammatory diseases (Alessandri et al., 2006; Mihai and Tervaert, 2010).

Although a number of previous publications have shown binding of SSc sera to the cell surface or to cytoplasmic targets of HUVEC this is the first direct demonstration that one of the antibodies produced is against the 90 kDa adhesion molecule ICAM-1. Interestingly, Ihn et al. previously identified a 90 kDa target for AECA from SSc patients although they showed that most binding was in the cytoplasm of HUVEC (Ihn et al., 2000).

We and others have previously demonstrated that cross-linking ICAM-1 on the surface of HUVEC or other endothelial cell tissue sources leads to pro-inflammatory signal transduction (review (Lawson and Wolf, 2009)). Here, we have recapitulated those findings after purifying anti-ICAM-1 antibodies from scleroderma patients, and shown that the purified antibodies bind to the cell surface of HUVEC, and after cross-linking induce an increase in ROS generation and VCAM-1 expression.

Several studies from this group and others have shown that whole serum with AECA activity or IgG fractions can similarly cause upregulation of adhesion molecules (Carvalho et al., 1996; Del Papa et al., 1996), whilst fibroblasts from SSc patients have been shown to produce more ROS than normal fibroblasts after cytokine stimulation (Sambo et al., 2001). Increased expression of ICAM-1 in skin and circulating in the serum of SSc patients has been reported (Ihn et al., 1997; Majewski et al., 1991), which could provide the initial stimulus to break B cell tolerance to this widely expressed adhesion molecule.

It is interesting to note that while significantly elevated levels of IgM anti-ICAM-1 antibodies were detectable in both lSSc and dSSc patients, elevated levels of IgG were only detectable in lSSc patients. Since lSSc is a much less severe form of scleroderma, with less likelihood of vascular involvement, one could postulate that these antibodies may have a protective or ameliorating effect on disease pathogenesis.

However, since we have also shown here that the IgG fraction is able to induce ROS production and VCAM-1 expression, which could be thought to be likely to lead to enhancement of vascular inflammation, elucidation of the exact nature of any protective effect requires further analysis. We have preliminary data (not shown) to demonstrate that the anti-ICAM-1 antibodies purified from scleroderma patients are able to activate MAPK pathways in HUVEC, in agreement with our previous findings with monoclonal anti-ICAM-1 antibodies (Lawson et al., 1999; Lawson et al., 2001). The pathways activated and the downstream biological effects are under further investigation. Our finding that ROS production after ligation of ICAM-1 by purified SSc antibodies with or without crosslinking was more sensitive to concentration effects than VCAM-1 expression (Fig. 3B), suggests that there are altered sensitivities in signal transduction pathways leading to VCAM-1 expression, or expression of other pro-inflammatory or protective genes activated via ROS, and will also be further investigated.

A limitation of use of HUVEC, or any non-transformed cells from a range of different individuals, is that the behaviour of different isolates may be altered. In this study the HUVEC isolates used to measure expression of VCAM-1 over a time course from 6 to 24 h appeared to be more responsive than those used to measure the concentration effects of the purified anti-ICAM-1 antibodies. This effect was also observed when comparing the response of these isolates to TNF (Fig. 4C and D). Interestingly, VCAM-1 expression after ICAM-1 cross-linking with patient purified antibodies at 1:100 dilution was always approximately 70% of that induced by TNF at 18 h in the same isolates.

In conclusion, our findings contribute to the wide body of evidence that AECA from SSc patients target specific endothelial antigens. In particular, we have shown here that SSc patient-derived anti-ICAM-1 antibodies cause pro-inflammatory activation of human endothelial cells, suggesting that they are not only a marker of disease but that they contribute to its progression.
